# A comparative study on approximate entropy measure and poincaré plot indexes of minimum foot clearance variability in the elderly during walking

**DOI:** 10.1186/1743-0003-5-4

**Published:** 2008-02-02

**Authors:** Ahsan H Khandoker, Marimuthu Palaniswami, Rezaul K Begg

**Affiliations:** 1 Department of Electrical & Electronic Engineering, The Universityof Melbourne, VIC 3010, Australia; 2 Biomechanics Unit, Centre for Ageing, Rehabilitation, Exercise and Sport, Victoria University, VIC 8001, Australia

## Abstract

**Background:**

Trip-related falls which is a major problem in the elderly population, might be linked to declines in the balance control function due to ageing. Minimum foot clearance (MFC) which provides a more sensitive measure of the motor function of the locomotor system, has been identified as a potential gait parameter associated with trip-related falls in older population. This paper proposes nonlinear indexes (approximate entropy (ApEn) and Poincaré plot indexes) of MFC variability and investigates the relationship of MFC with derived indexes of elderly gait patterns. The main aim is to find MFC variability indexes that well correlate with balance impairments.

**Methods:**

MFC data during treadmill walking for 14 healthy elderly and 10 elderly participants with balance problems and a history of falls (falls risk) were analysed using a PEAK-2D motion analysis system. ApEn and Poincaré plot indexes of all MFC data sets were calculated and compared.

**Results:**

Significant relationships of mean MFC with Poincaré plot indexes (SD1, SD2) and ApEn (r = 0.70, p < 0.05; r = 0.86, p < 0.01; r = 0.74, p < 0.05) were found in the falls-risk elderly group. On the other hand, such relationships were absent in the healthy elderly group. In contrast, the ApEn values of MFC data series were significantly (p < 0.05) correlated with Poincaré plot indexes of MFC in the healthy elderly group, whereas correlations were absent in the falls-risk group. The ApEn values in the falls-risk group (mean ApEn = 0.18 ± 0.03) was significantly (p < 0.05) higher than that in the healthy group (mean ApEn = 0.13 ± 0.13). The higher ApEn values in the falls-risk group might indicate increased irregularities and randomness in their gait patterns and an indication of loss of gait control mechanism. ApEn values of randomly shuffled MFC data of falls risk subjects did not show any significant relationship with mean MFC.

**Conclusion:**

Results have implication for quantifying gait dynamics in normal and pathological conditions, thus could be useful for the early diagnosis of at-risk gait. Further research should provide important information on whether falls prevention intervention can improve the gait performance of falls risk elderly by monitoring the change in MFC variability indexes.

## Background

Older population make up a large and increasing percentage of the population. As people grow older they are increasingly at risk of falling and consequent injuries. Approximately 30% of people over 65 fall each year, and for those over 75 the rates are higher. Between 20% and 30% of those who fall suffer injuries that reduce mobility and independence and increase the risk of premature death [1].

Human walking is a highly automated, rhythmic motor behaviour that is mostly controlled by subcortical locomotor brain regions. Gait analysis refers to the measurement and analysis of human walking patterns. One major aim of studying gait characteristics is to identify gait variables that reflect gait degeneration due to ageing with linkages to the causes of falls. This would help to undertake appropriate measures to prevent falls.

**Figure 1:**
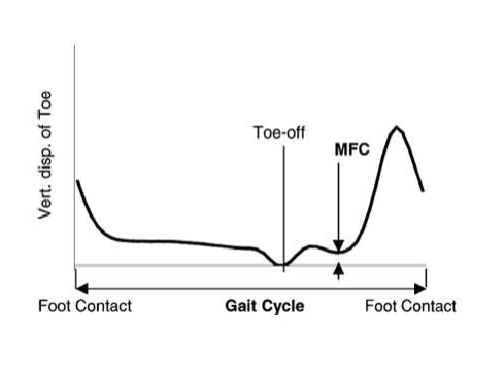
**Minimum foot clearance (MFC) during walking**. Vertical displacement of toe marker for one gait cycle (foot contact to foot contact) showing the occurrence of MFC event during mid swing (toe-off to foot contact) phase. (Reproduced with permission from Begg et al [11]). (copyright 2005 IEEE)

Minimum foot clearance (MFC) during walking (see Figure 1), which occurs during the mid-swing phase of the gait cycle, is defined as the minimum vertical distance between the lowest point under the front part of the shoe/foot and the ground, has been identified as an important gait parameter in the successful negotiation of the environment in which we walk. This is mainly because of the fact that during this MFC event, the foot travels very close to the walking surface (mean MFC = 1.29 cm) with a maximum forward velocity (4.6 m/s) [2]. The literature also suggests a decrease in MFC height (1.12 ± 0.50 cm) with ageing [3]. This small mean MFC value combined with its variability provides a strong rationale for MFC being associated with tripping during walking.

In our previous study [4], we studied the MFC variability and statistics for young and elderly females and described the changes of MFC central tendency and variability as one of the possible strategies by elderly individuals to minimize tripping. Analysis of linear statistics does not directly address their complexity and thus may potentially miss useful inherent information. Since the underlying mechanism involved in the human locomotor control has been reported to be mainly complex and nonlinear [5-7], the application of nonlinear technique seems appropriate. In this study, we, therefore, investigate the two types of nonlinear variability indexes (Approximate entropy and Poincaré plot indexes) of MFC to be able to perform a diagnostic function to distinguish walking patterns of elderly subjects with a history of balance impairments and falls from that of healthy peers.

Approximate entropy (ApEn), a mathematical approach to quantify the complexity and regularity of a system, has been introduced by Pincus [8], based on a novel systematic biological theory [8,9]. Such theory has suggested that healthy dynamic stability arises from the combination of specific feedback mechanisms and spontaneous properties of interconnected networks, and the weak connection between systems or within system is the mechanism of disease, which is characterized by an increased irregularity of the time series [9,10]. Therefore, ApEn was considered to provide a direct measurement of feedback and connection, and a low ApEn value often indicates predictability and high regularity of time series data, whereas a high ApEn value indicates unpredictability and random variation [9]. Previous studies [5] on the entropy of human gait in multiple scales discussed the scaling effect of entropy on various walking patterns, indicating the changes of multiscale entropy values with slow, normal and fast walking.

Poincaré plot is a geometrical representation of a time series into a Cartesian plane, where the values of each pair of successive elements of the time series define a point in the plot. Indexes derived from Poincaré plot of minimum foot clearance (MFC) were used to classify young-old gait types in our previous study [11].

With an aim to find a better marker of gait dynamics due to balance impairments, we apply ApEn analysis method to the MFC gait data obtained from elderly subjects with and without balance problem, and compare the results with those obtained using Poincaré plot indexes analysis.

## Methods

### MFC Gait Data

MFC data from 14 healthy elderly and 10 elderly with a history of falls (a history of falls was defined as an occurrence more than one fall) were taken from Victoria University's Biomechanics Unit database. Table 1 provides descriptive information for the two subject samples. All subjects (from local community and senior citizen clubs) undertook informed-consent procedures as approved by the Victoria University Human Research Ethics Committee. The detailed procedure for gait data collection has been described elsewhere [4]. In brief, foot clearance data were collected during steady state self-selected walking on a treadmill using the PEAK MOTUS 2D (Peak Technologies Inc, Centennial, USA) motion analysis system at 50 Hz. Two reflective markers were attached to each subject's left shoe at the fifth metatarsal head and the great toe. Each subject completed about 10 to 20 minutes of normal walking at a self-selected comfortable walking speed. The foot markers were automatically digitized for the entire walking task and raw data was digitally filtered using optimal cutoff frequency, which used a Butterworth filter with cutoff frequencies ranging from 4 to 8 Hz. The marker positions and shoe dimensions were used to predict the position of the shoe/foot end-point i.e., the position on the shoe travelling closest to the ground at the time when minimum foot clearance (MFC) occurs using a 2-D geometric model of the foot [4]. MFC for each gait cycle was calculated by subtracting ground reference from the minimum vertical coordinate during the mid-swing phase [4].

**Table 1:** Subject Characteristics, mean (± SD)

	**Healthy(n = 14)**	**Falls risk(n = 10)**
**Age (*years*)**	71.0 (± 2.1)	72.2 (± 3.1)
**Height (*cm*)**	170 (± 11)	166 (± 12)
**Weight (*kg*)**	63.2 (± 14.3)	66.9 (± 8.6)

### Estimation of ApEn of MFC

The algorithm for estimating ApEn of heart rate was first reported by Pincus [8]. We explain that approach as applied to MFC data. ApEn is defined as the logarithmic likelihood that the patterns of the data that are close to each other will remain close for the next comparison within a longer pattern. Given a sequence of total **N **numbers of MFC like MFC(1), MFC(2),........., MFC(N). To compute ApEn of each MFC data set, m-dimensional vector sequences p_m _(i) were constructed from the MFC series like [p_m _(1), p_m _(2),................, p_m _(N-m+1)], where the index i can take values ranging from 1 to N-m+1. If the distance between two vectors p_m _(i) and p_m _(j) is defined as |p_m _(j) - p_m _(i)|,

$${\text{C}}_{\text{i}}^{\text{m}}(\text{d})=\frac{1}{\text{N}-\text{m}+1}[\text{number of vectors such that }\left|{\text{p}}_{\text{m}}\text{(j)}-{\text{p}}_{\text{m}}\text{(i)}\right|<\text{d]}$$

Where **m **specifies the pattern length which is 2 in this study, **d **defines the criterion of similarity which has been set at 15% of the standard deviation of 400 MFC data which can produce reasonable statistical validity of ApEn [8,9]. Referring to theoretical analysis of ApEn statistics, Pincus and Goldberger [8] concluded that m = 2 and d = 10–25% of the standard deviation of N values (100–900 data points) will yield statistically reliable and reproducible results. C_i_^m^(d) is considered as the mean of the fraction of patterns of length *m *that resemble the pattern of the same length that begins at index i. ApEn is computed by using the following equation:

$$\text{ApEn}(\text{N},\text{m},\text{d})={\left(\text{N}-\text{m}+1\right)}^{-1}\sum_{\text{i}=1}^{\text{N}-(\text{m}-1)}\ln{\text{C}}_{\text{i}}^{\text{m}}(\text{d})-{\left(\text{N}-\text{m}\right)}^{-1}\sum_{\text{i}=1}^{\text{N}-\text{m}}\ln{\text{C}}_{\text{i}}^{\text{m}+1}(\text{d})$$

In our study, we use data set of 400 adjacent MFC data points. We divide the data set into smaller sets of length, i.e., m = 2. This amounts to 200 smaller sub sets. The next step is to determine the number of subsets that are within the criterion of similarity d = 15% of the standard deviation of 400 MFC points. Then we repeat the same process for the second subset till each subset is compared with the rest of the data set. This process computes ${\left(\text{N}-\text{m}+1\right)}^{-1}\sum_{\text{i}=1}^{\text{N}-\text{m}+1}\ln{\text{C}}_{\text{i}}^{\text{m}}(\text{d})$ part of equation (1) and N-m+1 = 400-2+1 = 399. We repeat the same process for m = 3. Approximate entropy is then calculated using equation (1).

### MFC Poincaré plots

**Figure 2:**
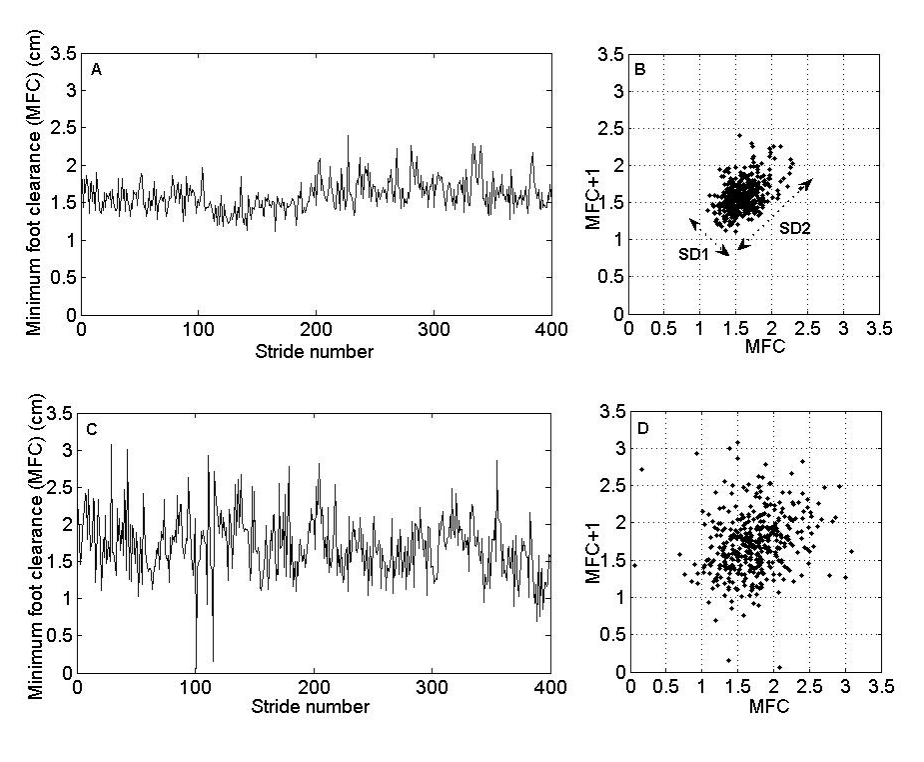
**MFC Poincaré plots**. Top panels show MFC time series from a healthy elderly subject (A) and its corresponding Poincaré plot (B). Bottom panels show MFC time series from an elderly subject with balance problem (C) and its corresponding Poincaré plot (D).

MFC data plots between successive gait cycles, i.e., between MFCn and MFCn+1 (see Figure 2B,D), known as MFC Poincaré plots [11], shows variability of MFC data and describes performance of the locomotor system in controlling the foot clearance at this critical event. Brennan et al. [12] provided mathematical expressions that relate each measure derived from Poincaré plot geometry to well-understood existing heart rate variability indexes. Using the method described by Brennan [12], these plots were used to extract indexes, such as length (SD2) and width (SD1) of the long and short axes of Poincaré plot images. Statistically, the plot displays the correlation between consecutive MFC data in a graphical manner. Points above the line of identity (y = x) indicate MFC data that are longer than the preceding MFC data point, and points below the line of identity indicate a shorter MFC distance than the previous. The MFC Poincaré plot typically appears as an elongated cloud of points oriented along the line-of-identity. The dispersion of points perpendicular to the line-of-identity reflects the level of short-term variability [12]. The dispersion of points along the line-of-identity is thought to indicate the level of long-term variability.

### Data analysis

All data were presented as mean ± SD. Associations between parameters and indexes were determined using Pearson's r. Student's (independent samples) t-test was used in order to compare the differences between the groups. In order to provide the relative importance of single index in discriminating two types of gait patterns, receiver-operating characteristics (ROC) curve analysis was used [13,14], with the areas under the curves for each measure represented by ROCarea. An ROCarea value of 0.5 means that the distributions of the variables are similar in both populations. Conversely, an ROCarea value of 1.0 means that the distributions of the variables of two populations do not overlap at all. A threshold value was applied such that any value below the threshold was assigned into a healthy category whereas a value equal to or above the threshold was assigned into falls risk category. True positive or sensitivity is defined as a measure of the ability of a single parameter to identify a falls risk gait, whereas false positive or specificity is a measure to detect healthy gait characteristics. ROC curve plots true positive against false positive as the threshold decision level is varied. The area under ROC curve was approximated numerically using the trapezoidal rules as described in [13,14]. The best accuracy, sensitivity and specificity obtained at a particular threshold for all features were also calculated with ROC areas. All data analyses were performed off-line, using custom software programs written for MATLAB (The Mathworks, Natick, MA).

### Surrogate data analysis

To prove any intrinsic relationship of locomotor control system with ApEn, we followed a method of surrogate data analysis introduced by Theiler et al. [15]. For each MFC series of falls risk subjects, 10 surrogate MFC series was obtained by randomly shuffling the original series. Each surrogate data sets had the identical MFC distribution (i.e., same mean, SD, and higher moments) as the original data sets and differed only in the sequential ordering of MFC series. Then the mean of the surrogate ApEn values were then calculated for the 10 surrogate data sets and compared to the ApEn of the original data set.

## Results

In order to compare the gait patterns of healthy elderly and falls-risk elderly, two representative examples of MFC time series and its corresponding Poincaré plots taken from each group have been presented in Figures 2A,B,C&D. Gait characteristics of a healthy elderly subject with mean MFC (= 1.56 ± 0.21 cm), and its corresponding Poincaré plot (Figure 2B) with indexes (SD1 = 0.31, SD2 = 0.5, SD1/SD2 = 0.63) and estimated ApEn (= 0.15) are visually different from the gait characteristics of falls-risk elderly subject with mean MFC (= 1.71 ± 0.41 cm), and its corresponding Poincaré plot (Figure 2D) with indexes SD1 = 0.72, SD2 = 0.92, SD1/SD2 = 0.79) and estimated ApEn (= 0.21). Table 2 shows the results from Student's t-test that average values of SD MFC, SD1 and SD2 in healthy elderly group were significantly different from those in the falls-risk elderly group (p < 0.05). It is interesting to note that difference between ApEn values in the two groups was highly significant (p = 0.0001).

**Table 2:** Mean ± standard deviation of parameters for healthy and falls-risk elderly subjects.

**Parameters**	**Heatlhy (n = 14)**	**Falls-risk (n = 10)**	**p value**
**Mean MFC**	1.65 ± 0.75	2.01 ± 0.51	**0.20004**
**SD MFC**	0.35 ± 0.13	0.48 ± 0.16	**0.0348**
**SD1**	0.51 ± 0.19	0.72 ± 0.25	**0.0309**
**SD2**	0.89 ± 0.32	1.15 ± 0.40	**0.0453**
**SD1/SD2**	0.64 ± 0.13	0.64 ± 0.12	**0.8912**
**ApEn**	0.13 ± 0.13	0.18 ± 0.03	**0.0001**

SD1 = Poincaré width, SD2 = Poincaré length, SD = standard deviation, ApEn = Approximate entropy.

Table 3 &4 show the Pearson correlation matrices among all tested indexes in the healthy elderly group and falls-risk elderly group.

**Table 3:** Correlation coefficients among measures of MFC in healthy elderly subjects

	**Mean MFC**	**SD MFC**	**SD1**	**SD2**	**SD1/SD2**	**ApEn**
**Mean MFC**	1	0.31	0.51	0.21	0.38	0.14
**SD MFC**		1	0.90***	0.99***	-0.36	-0.73**
**SD1**			1	0.81**	0.082	-0.68*
**SD2**				1	-0.50	-0.74**
**SD1/SD2**					1	0.38
**ApEn**						1

Correlation coefficients among mean MFC, Poincaré plot indexes (SD1, SD2, SD1/SD2) and ApEn of MFC in the healthy elderly subjects (n = 14). * p < 0.05 ** p < 0.01 *** p < 0.001 SD1 = Poincaré width, SD2 = Poincaré length, SD = standard deviation.

**Table 4:** Correlation coefficients among measures of MFC in falls risk elderly subjects

	**Mean MFC**	**SD MFC**	**SD1**	**SD2**	**SD1/SD2**	**ApEn**
**Mean MFC**	1	0.85***	0.70*	0.86**	-0.44	0.74*
**SD MFC**		1	0.90***	0.99***	-0.37	0.58
**SD1**			1	0.81**	0.06	0.49
**SD2**				1	-0.51	0.59
**SD1/SD2**					1	-0.28
**ApEn**						1

Correlation coefficients among mean MFC, Poincaré plot indexes (SD1, SD2, SD1/SD2) and ApEn of MFC in the falls-risk elderly subjects (n = 10).* p < 0.05 ** p < 0.01 *** p < 0.001 SD1 = Poincaré width, SD2 = Poincaré length, SD = standard deviation.

### Relationship between Poincaré plot indexes and mean MFC

**Figure 3:**
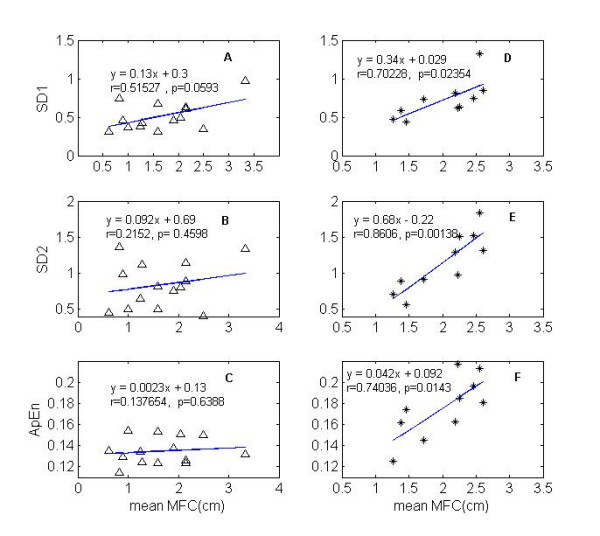
**Correlations among measures of MFC**. Panel A, B & C show the insignificant (P > 0.05) relationship of mean MFC with SD1 (A), SD2 (B) and ApEn (C) for the healthy elderly subjects (triangle) and panel D, E, & F show significant (P < 0.01) relationship of mean MFC with SD1 (D), SD2 (E) and ApEn (F) for the falls-risk elderly subjects (asterisk). r = Correlation coefficient. See tables 3 and 4 for details.

The correlation analysis shown in Figure 3 that there were significant relationship of mean MFC with SD1 and SD2 (r = 0.70, p < 0.05; r = 0.86, p < 0.01) in the falls-risk elderly group. On the other hand, no significant (p > 0.05) relationships were found in the healthy elderly group. An insignificant but inverse relationship was found between mean MFC and SD1/SD2 (r = -0.28, p > 0.05) in the falls-risk group (Table 3 &4).

### Relationship between ApEn and mean MFC

The correlation coefficient of mean MFC with ApEn in the falls-risk group (r = 0.74) was significantly (p < 0.05) higher than that in the healthy group (r = 0.14). Panel F in Figure 3 illustrates significantly positive correlation (r = 0.74, p < 0.05) between ApEn and mean MFC measures in the falls risk group, however, such correlation was absent in the healthy elderly group (panel C in Figure 3).

### Relationship between ApEn and Poincaré plot indexes

Correlation analysis also showed that ApEn was significantly inversely correlated with SD1 and SD2 (r = -0.68, P < 0.05; r = -0.74, p < 0.05) except SD1/SD2 (r = 0.38, p > 0.05) in the healthy elderly group. On the other hand, no significant (p > 0.05) but positive correlations were found between ApEn and SD1 & SD2 (r = 0.49, r = 0.59) in the falls-risk group. The relationship of ApEn with SD1/SD2 in falls-risk group was also insignificant but inverse (r = -0.28, p > 0.05).

### ApEn of surrogate MFC data

**Figure 4:**
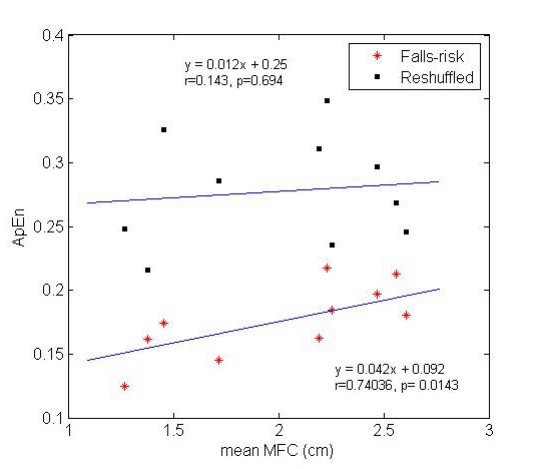
**Surrogate analysis**. Relationship of mean MFC with ApEn for the falls-risk elderly subjects (asterisk) and for the randomly shuffled MFC data sets of the same elderly subjects (solid square). Insignificant correlation (p > 0.05) was found in the reshuffled data sets. r = Correlation coefficient.

In order to test if the relationship of ApEn with mean MFC in falls-risk elderly subjects is truly due to any intrinsic characteristic of neural control of locomotor system, we considered the ApEn values of surrogate MFC data sets obtained by random shuffling described earlier in the methods. We compared the mean ApEn of surrogate MFC data with the ApEn values of the original MFC data. Figure 4 shows that significant positive relationship (r = 0.74, p < 0.05) abolished after shuffling (r = 0.14, p = 0.69). Mean ApEn values of surrogate MFC data in the falls-risk elderly group is 0.28 ± 0.04 (mean ± SD) which is significantly (p < 0.0001) higher than their original ApEn values.

### ROC curve analysis

**Figure 5:**
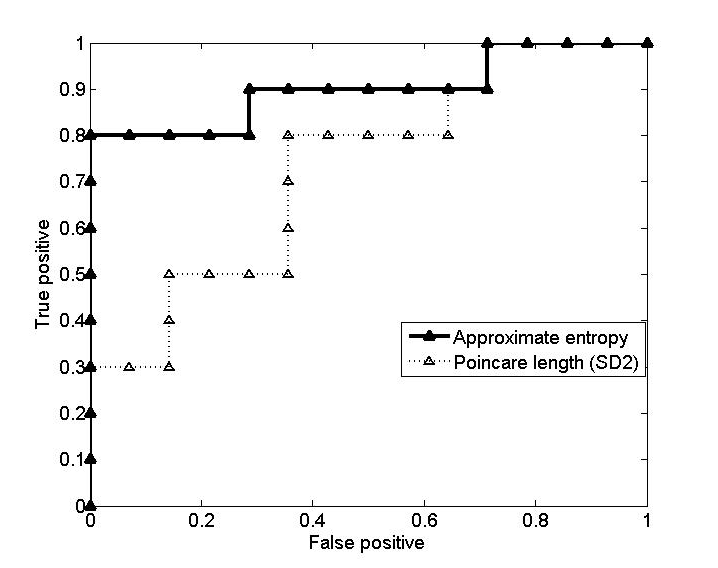
**ROC (receiver operating characteristics) curves**. ROC (receiver operating characteristics) curves showing true positive (sensitivity) and false positive rate (1-specificity) for various thresholds using Approximate entropy (ApEn) and length of the Poincaré plots (SD2) across 14 healthy elderly subjects and 10 falls-risk elderly subjects. Areas of ROC curves for ApEn and SD2 were 0.9 and 0.73 respectively. (Table 5)

Receiver Operating Characteristics (ROC) curves were used to characterize the quality of the single MFC indexes with respect to the identification task. Table 5 summarizes the classification accuracy, sensitivity, specificity and ROC areas calculated for each index. The larger area under ROC curve indicates better performance of that classifier. The largest ROC area (0.90) and highest classification performance (accuracy = 91.6%, sensitivity = 80% and specificity = 100%) were found for ApEn, whereas the lowest ROCarea (0.55) and lowest classification performance (accuracy = 62.5%, sensitivity = 70% and specificity = 57.14%) were for SD1/SD2 ratio. Figure 5 shows ROC curves for ApEn and SD2 in order to illustrate the comparative performance of ApEn and SD2 as a gait pattern identifier

**Table 5:** Classification performance

	**Mean MFC**	**SD MFC**	**SD1**	**SD2**	**SD1/SD2**	**ApEn**
**Accuracy**	75%	70.8%	70.8%	70.8%	62.5%	91.7%
**Sensitivity**	60%	70%	70%	30%	70%	80%
**Specificity**	85.7%	71.4%	71.4%	100%	57.14%	100%
**ROC area**	0.71	0.74	0.76	0.73	0.55	0.9

Classification performance (accuracy, sensitivity, specificity) and ROC areas for mean MFC, SD MFC, ApEn and Poincaré plot indexes (SD1, SD2, SD1/SD2) of MFC.

## Discussion

The results of this study highlight the implications of nonlinear variability indexes that have been utilized to characterize MFC signals of the elderly subjects during walking. Poincaré plot geometry and ApEn analysis of MFC gait data of elderly subjects provide useful information regarding identification of gait characteristics due to balance impairments in the elderly.

### MFC data and statistics

In this study, MFC data from steady-state gait have been used to characterize gait patterns. There are two major reasons for this. Firstly, MFC provides a more sensitive measure of motor function of the locomotor system compared to some gross overall kinematic descriptions of gait such as joint angular changes or stride phase times, secondly its close linkage with tripping falls [2,16]. Furthermore, long-term MFC data, as used in this study, are required so that variability indexes of MFC having long range correlation could be captured representative of the real gait performance. In our previous study [4] on MFC variability statistics for young/old gait patterns, we showed that MFC variability in the elderly is higher than that in the young subjects. Results from this study suggest that MFC variability in the healthy elderly is lower than that in the falls risk elderly. Higher mean MFC in the falls risk elderly group supports our previous findings [4] which showed that increasing the MFC height is one of the possible strategies used by elderly individuals to minimize tripping.

### MFC Poincaré plot indexes

Our results demonstrated that gait pathology due to balance impairments was reflected in altered MFC Poincaré plots (Figure 2D) and indexes extracted from these plots are effective in differentiating healthy and falls-prone gaits. Poincaré plot geometry was used in our earlier study for young-old gait pattern classification [11]. In this study, it has been extended to identifying elderly with a history of falls and balance problems. The pattern of MFC Poincaré plots and the increased range of SD1 and SD2 values are unique for particular type of gait abnormality like balance impairments. As both SD1 and SD2 are increased due to balance impairments (Table 3 &4) SD1/SD2 are not different between the two groups. Thus the indexes derived from this geometry may be considered as a characteristic parameter of diagnostic importance in clinical gait analysis. Nonlinear dynamics [17] considers the Poincaré plot as the two-dimensional (2-D) reconstructed MFC phase-space, which is a projection of the reconstructed attractor describing the dynamics of the locomotor system.

### ApEn analysis for MFC data

The importance of ApEn lies in the fact that it is a measure of disorder or randomness in the MFC signals. Higher ApEn values displayed in the falls-risk group might be an indication of randomness in the walking pattern of falls-risk elderly. On the other hand for healthy elderly subjects where MFC signals are more regular, ApEn has lower values. The value of ApEn reflecting the degree of irregularity, randomness and complexity of the MFC time series data, could therefore, indicate the degree of stability in the control of foot motion over the ground. In contrast, however, Goldberger [18] proposed that increased regularity of signals represents a 'decomplexification' of illness, citing numerous examples of illness states with increased regularity of rhythms. For example, Cheyne-Stokes respiration, Parkinsonian gait, loss of EEG variability, preterminal cardiac oscillations, neutrophil count in chronic myelogenous leukaemia and fever in Hodgkin's disease all exhibit periodic, more regular variation in the dynamics of disease states. In contrast to the 'decomplexification' hypothesis, Vaillancourt and Newell [19,20] noted increased complexity and increased approximate entropy in several disease states, including acromegaly and Cushing's disease, and hypothesized that disease may manifest with increased or decreased complexity, depending on the underlying dimension of the intrinsic dynamic (e.g. oscillating versus fixed point).

It is the first time that ApEn analysis has been used to characterize MFC signals. Therefore, values obtained in this study cannot be compared with other studies. However, a previous study involving stride interval gait time series, Costa et al [5] applied multi-scale entropy (MSE) for analysing gait with different speeds and studied the scaling effect on sample entropy for different walking rates. In that study, sample entropy (SampEn) in which self matches are excluded in the analysis, on multiple scales in normal spontaneous walking time series was found to be the highest value (i.e., highest complexity)when compared to slow and fast walking and also to walking paced by a metronome [5]. Although both SampEn and ApEn quantify the regularity of a time series, methods of calculation are different [21]. In our study, ApEn values of MFC in normal walking have been found to be higher in falls risk subjects than in healthy subjects. A principal advantage in the application of ApEn to biological signals is that ApEn statistics may be calculated for relatively short series of data which makes it a desirable application for routine diagnosis of possible gait impairment.

### Correlation analysis

Correlation analysis was designed to quantify the relationship of mean MFC with Poincaré plot indexes and ApEn values, and the relationships among these measures. Significantly positive correlations of mean MFC with SD1, SD2 and ApEn values in the falls risk subjects might indicate that MFC variability and its randomness significantly increase with an increase of mean MFC in falls risk gait. On the other hand, insignificant correlations (Table 3) in the healthy subjects indicate that MFC variability and its randomness insignificantly increase with an increase of mean MFC. Besides, it is also interesting to note that inverse correlations between SD1, SD2 and ApEn values were present in healthy subjects indicating that the more the variability the less the randomness (i.e. lower ApEn) in their gait (Table 3 &4). In contrast, an insignificant but positive correlations were found in falls risk subjects. One possible interpretation may be that higher SD1 and SD2 values, which correspond to higher short term and long term variability respectively, of falls risk subjects imply more random gait (i.e. higher ApEn) due to impaired balance control system. On the other hand, the increase of SD1 and SD2 values render more regular gait (i.e. lower ApEn) in the gait pattern of healthy elderly subjects. These results are interesting but it needs to be further investigated in a larger and more diverse sample of healthy and falls risk elderly adults.

### Surrogate data analysis

The use of surrogate data was aimed at destroying the underlying control mechanism and to increase the degree of randomness. Absence of correlation of mean MFC with ApEn and increased values of ApEn in the surrogate MFC data (shown in Figure 4) proved the presence of a particular locomotor control mechanism in the falls-risk elderly. Therefore, it could be inferred that MFC in the elderly walkers is not randomly executed from stride-to-stride rather it follows the fact that MTC output in such ageing gait is modulated by some other unknown mechanism which remains to be explored. These findings seem to support previous studies that have investigated complexity break down within both temporal and spatial [7] time series data amongst older adults and pathological groups.

### ROC curves and decision

Although both Poincaré plot indexes and ApEn were effective in discriminating the gait characteristics patterns, larger area under ROC curves for ApEn (Figure 5) suggested that ApEn could perform better than Poincaré plot indexes in classifying gait pattern. One possible reason why a nonlinear index like ApEn could be a more effective gait identifier might be that neural control mechanism of healthy human gait is nonlinear and hence, correlated with indexes derived from nonlinear analysis. This result could be useful in designing an automated gait pattern recognition model using nonlinear MFC variability indexes as input features.

### Future extensions

More research is needed to compare the prognostic value and clinical utility of the various statistical and new MFC variability measures before an ideal index can be introduced for clinical intervention purposes. Before the measurement of MFC variability can be considered to be of any clinical value, however, therapeutic interventions (e.g., exercise program to improve balance) are needed in the subjects who present with abnormal values (e.g., high ApEn values, higher MFC variability). Further validation should provide important information on whether falls prevention intervention can improve the gait performance of falls risk elderly by monitoring the change in linear and nonlinear MFC variability indexes. Different walking speeds may alter the MFC fluctuation magnitude which provides an alternative approach for future investigation of the relationship between ApEn and mean of MFC time series data.

## Conclusion

Early detection of gait pattern changes due to ageing and balance impairments using indexes derived from Poincaré plot geometry and ApEn analysis of MFC might provide the opportunity to initiate pre-emptive measures to be undertaken to avoid injurious falls. Also, such nonlinear index could potentially be used as gait diagnostic marker in clinical situation. Further investigation should be carried out to validate the associations of derived nonlinear MFC variability indexes with balance impairments in the falls risk subjects undergoing falls prevention intervention.

## Competing interests

The author(s) declare that they have no competing interests.

## Authors' contributions

RKB recruited subjects, managed data acquisition and participated to drafting of the manuscript. AHK and MP conceived the study, evaluated the data, performed data analyses and wrote the manuscript. All authors read and approved the final manuscript.
